# Efficacy and safety of sirolimus in the treatment of vascular malformations: A protocol for systematic review and meta-analysis

**DOI:** 10.1097/MD.0000000000022596

**Published:** 2020-10-02

**Authors:** Jianyong Dong, Deting Han, Desheng Wang, Huijun Lu, Xiaoliang Wang

**Affiliations:** a Gaoxin Branch of Jinan Stomatological Hospital; b Shandong University of Traditional Chinese Medicine, Jinan, China.

**Keywords:** protocol, sirolimus, systematic review, vascular malformation

## Abstract

**Background:**

The pathophysiologic of vascular malformations is still unclear, and the treatment of vascular malformations is a challenge. With improvement in the understanding of pathogenesis of vascular malformations, sirolimus has been a promising and effective treatment. As so far, there is absent convincing evidence to confirm the efficacy of sirolimus for vascular malformations. The purpose of this study was to evaluate the effectiveness and safety of sirolimus in the treatment of vascular malformations.

**Methods:**

The literatures about the management of vascular malformations with sirolimus would be searched from databases of MEDLINE, EMBASE, PubMed, Web of Science, Clinicaltrials.org., Cochrane Library, China Biology Medicine Database (CBM), Wan Fang Database, China National Knowledge Infrastructure Database (CNKI), and VIP Science Technology Periodical Database. We will search each database from inception or 1995 to August 20, 2020. Two researchers worked independently on literature selection, data extraction and quality assessment. The efficacy and safety of sirolimus in the treatment of vascular malformations were the main outcomes. Adverse events after sirolimus were evaluated as the secondary outcomes. The included studies will be analyzed by Review Manager 5.3. If the results are applicable, meta-analysis would also be performed.

**Results:**

The study will evaluate the efficacy and safety of sirolimus in the treatment of vascular malformations based on current evidence.

**Conclusion:**

The conclusion of this study will provide more reliable, evidence-based data for the use of sirolimus in the treatment of vascular malformations.

**PROSPERO registration number:**

CRD42020167881.

## 1 Introduction

Vascular malformations(VMs), characterized by the abnormal development or growth of blood and/or lymphatic vessels, can be subdivided into capillary, venous, lymphatic, arterio-venous, and combined malformations.^[1]^ VMs are always present at birth, increase in size, and never regress spontaneously, and may cause clinical problems such as disfigurement, acute and chronic pain, infections, coagulopathy, bleeding, thrombosis, functional impairment, and even death.^[2]^ Treatment of VMs is complex, including surgical excision, laser, sclerotherapy, and embolization. There is no consensus for management of vascular malformations.^[3]^

Further research in the genetic and pathophysiologic origin of VMs demonstrated that VMs caused by abnormal signaling within vascular endothelial cells.^[4]^ The phosphatidylinositol 3-kinase(PI3K)-protein kinase B(AKT)-mammalian target of rapamycin (mTOR) pathway plays a key role in the pathogenesis of VMs.^[5]^ The PI3K/AKT/mTOR pathway is implicated in many cellular processes, such as cell-cycle regulation, proliferation, protein synthesis, and cell survival.^[6]^ The discovery of the pathogenic involvement of the PI3K-AKT-mTOR pathway in VMs make the treatment of these lesions with targeted drug possible. Sirolimus is an immunosuppressive agent that inhibits the action of mTOR. Sirolimus has emerged as one of the treatments for VMs.^[7]^ For some complicated VMs which are refractory to the traditional treatment, sirolimus had beneficial effect.[^8^,^9^,^10^] Off-label use of mTOR inhibitors was reported to be efficient in different types of VMs, with heterogeneous outcomes.[^11^,^12^,^13^] But promising results from several phase I and II prospective studies[^14^,^15^,^16^] and from retrospective case series have led to a phase III clinical trial (VASE, EudraCT Number: 2015–001703-32) that is currently underway in Europe. The objective of this study was to systematically evaluate the data published about the efficacy and safety of sirolimus in the treatment of VMs and to compare the efficacy rates of sirolimus for the different kinds of VMs. It is our hope that the study could provide useful treatment reference for the use of sirolimus for the treatment of VMs.

## 2 Methods

The protocol has been registered in PROSPERO (CRD42020167881) at https://www.crd.york.ac.uk/PROSPERO/. This protocol will be reported according to the Preferred Reporting Items for Systematic Reviews and Meta-Analyses Protocols (PRISMA-P) statement, and the systematic review will be reported with the PRISMA statement.^[17]^ Ethical approval is not required as this study is based on aggregate data and will not involve humans.

### 2.1 Data source

#### 2.1.1 Electronic search database and approach

The data and information will be retrieved from the databases of MEDLINE, EMBASE, PubMed, Web of Science, Clinicaltrials.org., Cochrane Library, China Biology Medicine Database (CBM), Wan Fang Database, China National Knowledge Infrastructure Database (CNKI), and VIP Science Technology Periodical Database. The search terms included “vascular malformations,” “venous malformations,” “arteriovenous malformation,” “lymphatic malformation, “sirolimus,” “rapamycin,” and the Chinese key words corresponding to the words above. We only included articles published since 1995 when the International Society for the Study of Vascular Anomalies (ISSVA) classification system was generally accepted.^[18]^ The search will be performed for studies from inception or 1995 to August 20, 2020.

#### 2.1.2 Search strategy

The strategy will be created according to the Cochrane handbook guidelines. The established search strategy for PubMed was displayed in Table 1. The right retrieval strategy would be adapted for different databases.

**Table 1:**
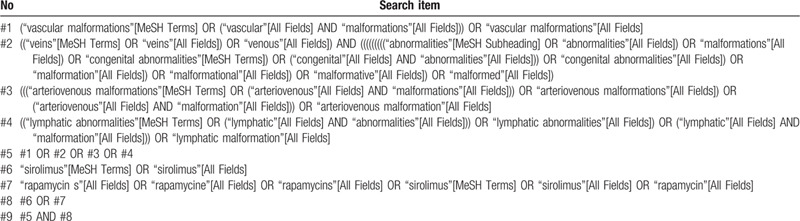
Details of the search strategy for PubMed.

### 2.2 Included and excluded criteria

Inclusion criteria were all original reports (randomized controlled trial, prospective and retrospective case series, and case reports) describing treatment with sirolimus (topical or oral) in VMs, without age and sex restrictions. The language was restricted to English and Chinese.

Duplicated publications, reports with insufficient information, and abstracts and revision articles were excluded. Patients treated with a combination of therapies were also excluded.

### 2.3 Outcomes

#### 2.3.1 Primary outcome indicator

The primary outcome of this study is the efficacy of sirolimus. Response was defined as follows: Complete response, defined as a complete; Partial response, defined as a reduction of ≥20% in size of the vascular lesion disappearance of the lesion; Absence of response, defined as a progressive disease or disease stability.

#### 2.3.2 Secondary outcome indicators

Secondary outcomes are adverse events after sirolimus. The commonly side effects of sirolimus included stomatitis, headache, infections, acne, and hyperlipidemia. Adverse events were assessed according to the Common Terminology Criteria for Adverse Events version 3.0.

### 2.4 Selection of studies and data extraction

**Figure 1:**
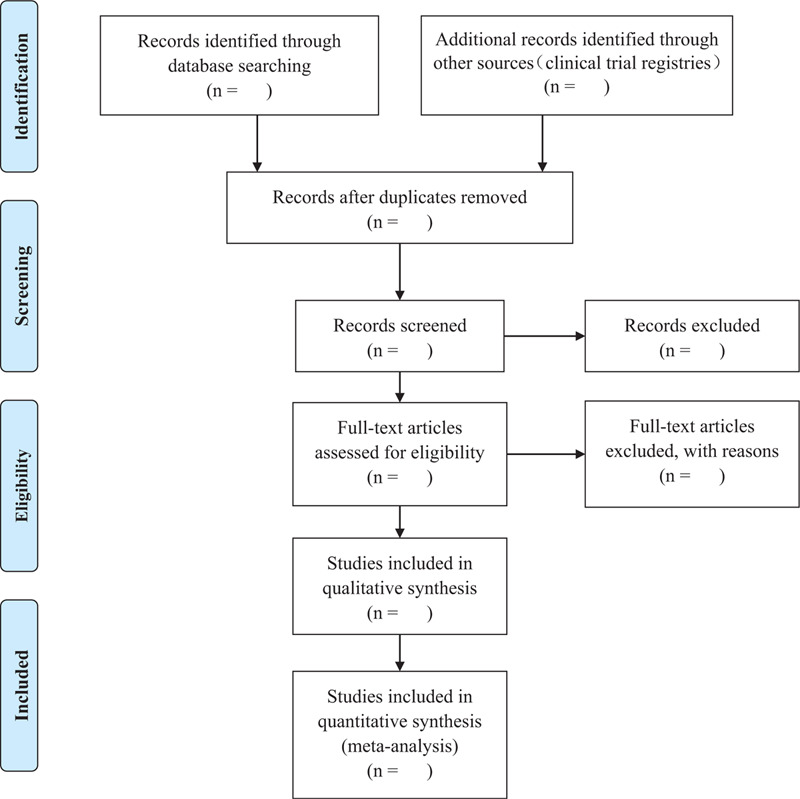
Flow diagram of systematic literature search and study selection.

Two well-trained reviewers (Jianyong Dong, Deting Han) searched the databases, respectively. The PRISMA flow diagram for study selection is shown in Figure 1. They extra data and information separately with a pre-designed data extraction form, including the publication information, type of vascular malformations, characteristics of patients, interventions, outcomes, study design, adverse events, and other detailed information. Any disagreement will be discussed and resolved with the third professional reviewer (Xiaoliang Wang).

### 2.5 Risk of bias assessment

Each of the 2 well-trained reviewers will evaluate the risk of bias by himself. If it cannot be determined, a discussion will be carried out among all reviewers. The results were presented as a risk of bias graph and risk of bias summary using the Cochrane Collaborations software program Review Manager version 5.3 for Windows.

### 2.6 Assessment of heterogeneity

Statistical heterogeneity of included studies would be assessed by the *I* ^*2*^ test and Chi-Squared test. If *I* ^*2*^ < 50%, *P* > .1, there is no statistical heterogeneity and fixed effect model will be chosen to synthesize the data. If *I* ^*2*^ ≥ 50%, *P* < .1, heterogeneity exists among studies, and the random effect model is used.

### 2.7 Data analysis and synthesis

If the studies are homogeneous without heterogeneity, meta-analysis can be performed by software Review Manager 5.3, otherwise we only can perform a general descriptive analysis.

### 2.8 Subgroup analysis

If there are significant clinically and statistically heterogeneous in the researches, subgroup analysis will be performed according to the types of vascular malformations.

### 2.9 Sensitivity analysis

Sensitivity analysis is conducted to test the quality of the meta-analysis result. Remove the studies with high risk of bias and merge the data to identify the factors for the causation of heterogeneity.

### 2.10 Grading the quality of evidence

The quality of evidence will be assessed on the basis of guidelines of the Grading of Recommendations Assessment, Development, and Evaluation (GRADE) and divided into 4 level: very low, low, moderate, or high.^[19]^

## 3 Discussion

Vascular malformations were divided into 4 groups: simple, combined, those of major named vessels (lymphatics, veins, arteries), and those associated with other anomalies based on the biology and genetics of lesions. The new classification systems for VMs help to improve the management of lesions. But the management of VMs was still challenging and required a multidisciplinary approach, especially for the complex lesions. Surgical or interventional procedures to destroy or to remove abnormal vessels were the usually treatment choices. In some cases, these methods made a poor effect and invasive with associated risks of complications and recurrence rates. With the understanding of pathophysiology of VMS, the specific somatic mutations and the potential molecular targets were identified.^[20]^ It made the specific target medical therapies of lesions possible. The somatic mutation in PI3K/AKT/mTOR signaling pathway discovered in VMs, which is critical to cell growth and proliferation.[^21^,^22^] The new discoveries are the foundation for the treatment of VMs with sirolimus. Positive response with limited toxicity of sirolimus in treatment of VMs were reported in numerous reports,[^11^,^23^,^24^] but large prospective clinical trials are limited. We do the study to evaluate the efficacy and safety of sirolimus in the treatment of VMs. But the guideline of sirolimus treatment for VMs is lacking, and the information about the long-term toxicities of sirolimus remains unknown. More prospective clinical trials are underway to investigate the safety and efficacy of sirolimus in the treatment of VMs.

## Author contributions

**Conceptualization:** Xiaoliang Wang.

**Data curation:** Deting Han, Huijun Lu, Desheng Wang.

**Formal analysis:** Deting Han, Huijun Lu, Desheng Wang.

**Funding acquisition:** Jianyong Dong.

**Methodology:** Deting Han, Huijun Lu, Desheng Wang.

**Project administration:** Deting Han, Xiaoliang Wang.

**Resources:** Deting Han, Desheng Wang.

**Software:** Deting Han, Desheng Wang.

**Supervision:** Xiaoliang Wang.

**Validation:** Xiaoliang Wang.

**Visualization:** Xiaoliang Wang.

**Writing – original draft:** Jianyong Dong, Xiaoliang Wang.

**Writing – review & editing:** Jianyong Dong, Xiaoliang Wang.
